# MAGE-A protein and MAGE-A10 gene expressions in liver metastasis in patients with stomach cancer

**DOI:** 10.1038/sj.bjc.6604476

**Published:** 2008-07-01

**Authors:** S Suzuki, K Sasajima, Y Sato, H Watanabe, T Matsutani, S Iida, M Hosone, T Tsukui, S Maeda, K Shimizu, T Tajiri

**Affiliations:** 1 Department of Surgery, Tama-Nagayama Hospital, Nippon Medical School, Tama, Tokyo, Japan; 2 Department of Molecular Diagnostics, School of Allied Health Science, Kitasato University, Sagamihara, Kanagawa, Japan; 3 Department of Surgery, Nippon Medical School, Bunkyo-Ku, Tokyo, Japan; 4 Department of Pathology, Tama-Nagayama Hospital, Nippon Medical School, Tama, Tokyo, Japan; 5 Department of Gastroenterology, Tama-Nagayama Hospital, Nippon Medical School, Tama, Tokyo, Japan

**Keywords:** MAGE-A protein, MAGE-A10 mRNA, highly sensitive *in situ* hybridisation, stomach cancer, liver metastasis, active immunotherapy

## Abstract

Tumour samples from 71 patients with stomach cancer, 41 patients with liver metastasis (group A) and 15 patients each in stages II–IV (group B) and stage I (group C) without liver metastasis were analysed. MAGE-A protein expression was evaluated by immunohistochemistry using a 6C1 monoclonal antibody and MAGE-A10 mRNA expression was detected by highly sensitive *in situ* hybridisation using a cRNA probe. Expressions of MAGE-A protein and MAGE-A10 mRNA in group A were detected in 65.9 and 80.5%, respectively. Both protein and gene showed significantly higher expression in group A than those in groups B (6.7, 26.7%) and C (0, 0%) (*P*=0.0003, *P*=<0.0001, respectively). MAGE-A10 mRNA expression in liver metastasis was found in eight (88.9%) out of nine patients. The concordant rate between MAGE-A family protein expression and MAGE-A10 mRNA expression in the primary sites was 81.7% (*P*<0.0001). MAGE-A10 gene expression was associated with reduced survival duration. The results of this study suggest that MAGE-A10 is a possible target in active immunotherapy for advanced stomach cancer.

Stomach cancer is one of the most common worldwide malignant neoplasms (Dicken *et al*, 2005). Although its incidence is decreasing, it is the second most common cancer in Japan after lung cancer and contributes to the burden of cancer death (Inoue and Tsugane, 2005). The presence of distant metastasis is an independent prognostic factor (Siewert *et al*, 1998). In particular, patients with liver metastasis had shown a poor prognosis in spite of combined therapy (Sarela *et al*, 2006). Therefore, further study to understand the molecular changes associated with stomach cancer especially when tumour progresses needs to be undertaken to introduce treatment strategies. Furthermore, the mechanism of liver metastasis should be elucidated, as it may present a clue to predict and subsequently treat patients with liver metastasis.

It has been reported that MAGE-A family genes located at chromosome Xq28 are expressed in malignant tumours, whereas they are not expressed in adult tissue except the testis and placenta. Of the MAGE-A family, MAGE-A1, -A3, -A6 and -A10 encode tumour antigens recognised by autologous cytotoxic T cells (CTL) (De Plaen *et al*, 1994). A CTL clone that could lyse autologous melanoma cells significantly produced tumour necrosis factor on stimulation with HLA-A2 MAGE-A10-expressing cells (Huang *et al*, 1999). Only CTL recognising the antigenic peptide with high efficiency can lyse the tumour cells expressing the cancer/testis (CT) antigens. Thus, these antigens may be suitable targets for active immunotherapy in malignant tumours such as melanoma (Dutoit *et al*, 2001, 2002; Ayyoub *et al*, 2003).

In stomach cancer, MAGE-A protein expression was detected in only 15.8% of the samples, and correlated with lymph node metastasis, advanced stage of the disease and a worse prognosis (Jung *et al*, 2005). However, MAGE-A protein was expressed at lower levels in metastatic lymph nodes from stomach cancer than in the primary lesions (Sadanaga *et al*, 1999). The frequency of MAGE-A1, -A2 and -A3 gene expression was reported to be 41, 31 and 38% in the primary lesion, respectively (Inoue *et al*, 1995). About 74% of the tumours expressed at least one CT antigen, most frequently MAGE-A3, -A4 and NY-ESO-1. However, MAGE-A gene expression did not correlate with clinicopathologic findings of the tumours (Wang *et al*, 2004). MAGE-A10 expression was reported to be low in the primary lesion and had no correlation with the clinicopathology of the tumours (Li *et al*, 1997). There have been no reports on the expression of the MAGE-A10 gene in metastatic lesions in patients with stomach cancer.

In this study, the MAGE-A family protein expression was detected by immunohistochemistry using the mouse monoclonal antibody 6C1, which cross-reacted with MAGE-A1, -A3, -A4, -A6, -A10 and -A12 (Rimoldi *et al*, 2000). Furthermore, to clarify the distribution of MAGE-A10 mRNA expression in stomach cancer tissue, the highly sensitive *in situ* hybridisation (ISH) using cRNA probes that enabled us to detect even the low-copy gene expression in clinical samples was performed. Our objective was to assess the significance of MAGE-A protein and MAGE-A10 gene expressions in the progression of stomach cancer and to elucidate suitable patients for active immunotherapy using MAGE-A10 peptide.

## Materials and methods

### Tumour samples

Formalin-fixed and paraffin-embedded tissues were obtained from a series of 71 patients who had undergone gastrectomy for stomach cancer at the Tama-Nagayama Hospital, Nippon Medical School. In five unresectable cases, biopsy specimens were used. The patients were divided into three groups. Group A consisted of 41 patients with liver metastasis occurring within 3 years after the operation: synchronous metastasis (stage IV) in 23 cases and metachronous metastasis (stages I–IV) in 18 cases. In group A, nine hepatectomised specimens (synchronous in six, metachronous in three) were used, in addition to the primary sites. Groups B and C consisted of 15 patients each with stages II–IV and stage I without liver metastasis. The final stage and histopathologic findings are defined according to the Japanese Classification of Gastric Carcinoma (Japanese Gastric Cancer Association, 1998). Patients' characteristics and pathologic findings are listed in Table 1. Informed consent was obtained from all patients. The study was approved by the Institutional Review Board of Tama-Nagayama Hospital, Nippon Medical School.

### Immunohistochemistry

The sections were deparaffinised in xylene and hydrated through descending ethanol series and water. Antigen retrieval was performed with the DAKO Target Retrieval Solution (DAKO, Glostrup, Denmark) for 40 min at 98°C. Endogenous peroxidase blocking was performed with 0.3% hydrogen peroxide in methanol. After incubation with the mouse monoclonal anti-MAGE-A antibody 6C1 (Neo-Markers, Fremont, CA, USA) for 30 min at room temperature, DAKO ENVISION Plus detection was applied for 30 min at room temperature. Then, diaminobenzidine was dropped as a chromogen. The slides were counterstained with haematoxylin and mounted.

### *In situ* hybridisation

#### Preparation of the cRNA probes

Total RNA was extracted from LCN1 cells derived from neuroendocrine carcinoma of the lung (Jiang *et al*, 2004) with Isogen (Nippon Gene, Tokyo, Japan) according to the manufacturer's instructions and reverse transcribed with the First Strand cDNA Synthesis Kit for RT–PCR (Roche Diagnostics, Mannheim, Germany). Primer sequences used in this study against MAGE-A10 mRNA have been described previously (Peng *et al*, 2005). Digoxigenin (DIG)-labelled sense and antisense cRNA probes were generated by T7 RNA polymerase promoter region-tailed PCR followed by *in vitro* transcription with T7 RNA polymerase using a DIG *in vitro* transcription kit (Roche Diagnostics) described in detail previously by Tanizaki *et al* (2006).

#### Highly sensitive *in situ* hybridisation

The ISH was carried out as described previously (Yoshikawa *et al*, 2007). In brief, deparaffinised 4-*μ*m-thick sections were treated with 10 *μ*g ml^−1^ proteinase K (Roche Diagnostics) for 20 min at 37°C. The sections were post-fixed in 4% paraformaldehyde and treated with 0.2 N HCl and 0.25% acetic anhydride in 0.1 M tri-ethanol amine (pH 8.0) for 10 min each. After treatment with 3% hydrogen peroxide for 60 min, the sections were dehydrated and air-dried. A 50 *μ*l portion of the hybridisation mixture (mRNA *In-situ* Hybridization Solution) (DAKO) with 10 ng sense or antisense cRNA probe was loaded onto each section and hybridised for 16–18 h at 50°C. After hybridisation, the sections were washed in 2 × standard sodium citrate (SSC)/50% formamide for 30 min at 55°C and treated with 10 *μ*g ml^−1^ RNase A (Roche Diagnostics) for 30 min at 37°C and stringently washed with 2 × SSC, 0.2 × SSC and 0.1 × SSC for 20 min each at 55°C. After being placed into 0.01 M Tris-HCl pH 7.5, 0.3 M NaCl and 0.1% Tween 20 (TBS-2T) three times for 5 min each, and in 0.5% casein/0.01 M Tris-HCl pH 7.5 and 0.15 M NaCl for 10 min, the sections were reacted with 400 times diluted horseradish peroxidase (HRP)-conjugated rabbit anti-DIG Fab′ fragmented polyclonal antibody (DAKO), 0.07 *μ*mol l^−1^ biotinylated tyramide and 500 times diluted HRP-conjugated streptavidin (DAKO) for 15 min each. Finally, the sections were visualised with DAB solution (DAB Substrate Kit) (DAKO) and counterstained with Mayer's haematoxylin. Thereafter, sections were mounted with Pristine Mount (Invitrogen, Carlsbad, CA, USA).

### Scoring of immunohistochemistry and *in situ* hybridisation

Evaluation of tumour cells in each sample was scored as 0, 1, 2 or 3, corresponding to absent, weak, moderate or intense staining, respectively. The intensity of the cells scoring moderate or higher was judged as positive. The tissues consisting of more than 30% positive tumour cells were considered significant.

### Statistical analysis

Fisher's exact test, Kaplan–Meier survival analysis and log-rank test for univariate analysis were performed using the Statview 5.0 software (Abacus System, Berkeley, CA, USA). *P-*values<0.05 were considered significant.

## Results

### MAGE-A protein and MAGE-A10 mRNA expression in the primary lesion

**Figure 1:**
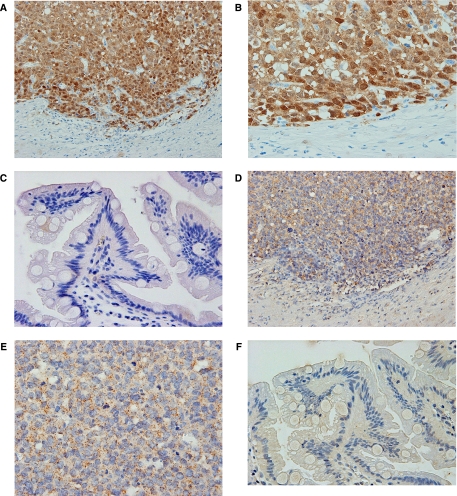
A case with synchronous liver metastasis (case 13). At the primary site, (**A**) MAGE family proteins were observed in the nuclei of tumour cells ( × 80). (**B**) High-power view of (**A**). They were dominantly and homogeneously localised in the nuclei of tumour cells ( × 200). (**C**) At the normal gastric mucosa, MAGE family proteins were not observed ( × 80). (**D**) MAGE-A10 mRNA signals were coincidently observed in MAGE family protein-positive tumour cells ( × 80). (**E**) High-power view of (**D**). They were abundant in the cytoplasm of tumour cells ( × 200). (**F**) At the normal gastric mucosa, MAGE-A10 mRNA signals were not found ( × 80).

The incidence of MAGE-A protein expression was 65.9% (27 cases) in group A, 6.7% (1 case) in group B and 0% in group C. Although both nucleus and cytoplasm were stained against 6C1 in most of the cases, in some cases the nucleus was dominantly stained (Figure 1A and 1B). No positive staining was found in any of the non-cancerous tissues (Figure 1C). Of 28 cases positive for 6C1, the distribution of positively stained cells showed a diffuse pattern in 67.9% (19 cases) and a focal pattern in 32.1% (9 cases). The incidence of MAGE-A protein in group A was significantly higher than that in groups B and C (*P*=0.0001 and *P*<0.0001, respectively).

The incidence of MAGE-A10 mRNA expression was 80.5% (33 cases) in group A, 26.7% (4 cases) in group B and 0% (none) in group C. No positive staining was observed with the sense cRNA probe. Figure 1D and 1E presents a case with MAGE-A10 mRNA expression in the primary site (case 13). No positive staining was observed in any of the non-cancerous tissues (Figure 1F). Further, no positive staining was observed with the sense cRNA probe. Significant differences were detected between group A and groups B and C (*P*=0.0003 and *P*<0.0001, respectively). MAGE-A10 mRNA expression with liver metastasis was more frequent in 87.0% of synchronous metastasis compared with 72.2% of metachronous metastasis. However, no statistical significance was observed. The association of the expression of MAGE-A protein and MAGE-A10 mRNA is listed in Table 2. Of 37 cases with MAGE-A10 mRNA expression, 70.3% (26 cases) were positive for MAGE-A protein. The rates of concordance between MAGE-A protein expression and MAGE-A10 mRNA expression were 75.6% in group A, 80.0% in group B and 100% in group C. Totally, the rate of concordance was 81.7% (*P*<0.0001).

### MAGE-A protein expression and MAGE-A10 mRNA expression in liver metastasis

**Figure 2:**
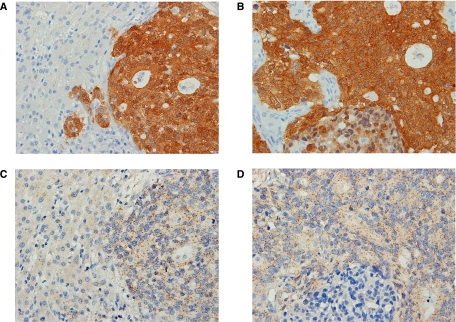
A case with synchronous hepatic metastasis (case 7). At the hepatic metastasis, (**A**) MAGE family proteins were observed in both nuclei and cytoplasm of tumour cells. No positive staining was observed in adjacent normal liver tissues ( × 80). (**B**) High-power view of (**A**). Intense and homogeneous staining was observed in almost all tumour cells ( × 200). (**C**) MAGE-A10 mRNA signals were coincidently observed in MAGE family protein-positive tumour cells ( × 80). (**D**) High-power view of (**C**). They were abundant in the cytoplasm of tumour cells ( × 200).

In nine cases of hepatectomy or liver biopsy for metastatic lesions, positive expressions were detected in seven (77.8%) for MAGE-A protein and eight (88.9%) for MAGE-A10 mRNA (Table 3). Two cases in the positive MAGE-A10 mRNA group were negative for 6C1 expression (cases 16 and 21). Both MAGE-A protein and MAGE-A10 mRNA expressions in hepatic tissue with a synchronous liver metastasis (case 7) are shown in Figure 2.

### MAGE-A protein expression and MAGE-A10 mRNA expression in alpha fetoprotein-producing stomach cancer

Of five cases with alpha fetoprotein (AFP) producing stomach cancer proved by serum concentration and immunohistochemistry, MAGE-A protein expression and MAGE-A10 mRNA expression were found in three cases (60.0%) and five cases (100%), respectively. Five cases of positive MAGE-A10 mRNA expression were intensely and diffusely stained in the tumour cells. Figure 1 presents a case with AFP-producing stomach cancer expressing MAGE-A protein and MAGE-A10 mRNA (case 13).

### Survival analysis

**Figure 3:**
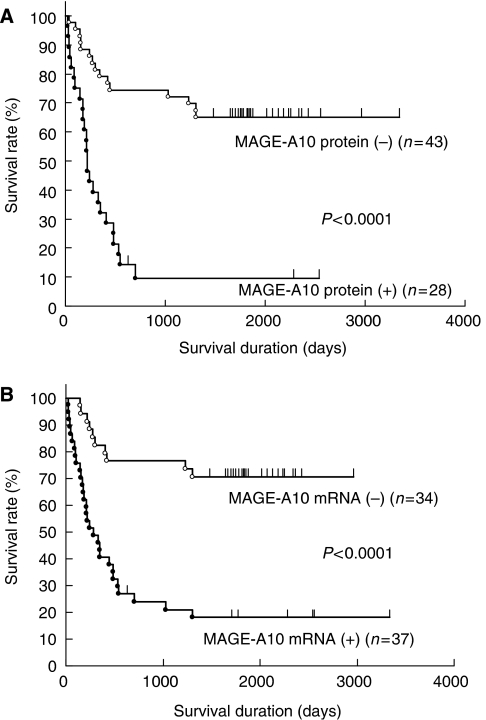
Relationship between MAGE-A family protein (**A**) and MAGE-A10 mRNA (**B**) expression and survival duration in all cases.

Kaplan–Meier analysis revealed that group A had significantly shorter survival duration than groups B and C (*P*<0.0001, each). A 5-year survival rate was 6% in group A, 87% in group B and 100% in group C. Patients with MAGE-A protein and MAGE-A10 gene expression had poor outcome (*P*<0.0001, each) (Figure 3A and 3B). Only three patients with MAGE-A10 gene expression survived more than 5 years.

## Discussion

In this study, we have analysed MAGE-A protein expression by immunohistochemistry using a monoclonal 6C1 antibody and MAGE-A10 gene expression by ISH using a cRNA probe in stomach cancer patients with and without liver metastasis. This is the first report, to the best of our knowledge, that provides a new insight into the expressions of the MAGE-A protein and MAGE-A10 gene in patients with stomach cancer. The expression of both was significantly higher in patients with liver metastasis than in patients without liver metastasis. We also found that the MAGE-A10 gene was frequently expressed in the primary and metastatic lesions and was associated with a poor outcome.

In stomach cancer, MAGE-A protein expression was associated with the stage and a worse prognosis, but not with distant organ metastasis (Jung *et al*, 2005). MAGE-A1 gene expression has been reported to be observed in the early stage of stomach cancer (Katano *et al*, 1997). However, Honda *et al* (2004) reported that demethylation in MAGE-A1 and -A3 promoters was much higher in advanced clinical stages. Distant metastasis is an independent prognostic factor in patients who have undergone surgical resection (Siewert *et al*, 1998). Recurrence after complete resection of stomach cancer occurs within 2 years and is fatal. Furthermore, there was recurrence in distant sites in half of the patients and liver metastasis was most frequent (D'Angelica *et al*, 2004). In this study, most cases with both synchronous and metachronous liver metastasis expressed the MAGE-A protein and MAGE-A10 gene in both primary and metastatic lesions. Although the total number was small in our series, two stage I patients who developed metachronous liver metastasis expressed MAGE-A10 mRNA in the primary lesions. It might be a predictive marker for metachronous liver metastasis even in low-stage stomach cancers. On the other hand, Alves *et al* (2007) reported that MAGE-A10 mRNA expression was found in only 2 and 0% of the cases of primary lesion and liver metastasis in colon cancer, respectively. We assume that this discrepancy might be due to the differences in the regulation of MAGE-A gene expression and the clinical outcome of liver metastasis between these organs. These results emphasised that expression of the MAGE-A genes might play an important role in the progression of stomach cancer.

AFP-producing stomach cancer is highly malignant and has a poor prognosis as the recurrent rate following curative resection is high (Adachi *et al*, 2003). Amemiya *et al* (2005) reported that c-Met overexpression was frequently detected in AFP-producing stomach cancer, whereas Kataoka *et al* (2001) reported that the absence of ATBF1 gene expression is responsible for AFP gene expression. Recently, Cho *et al* (2007) reported that five of the eight cancers with AFP expression showed genetic alterations of the ATBF1 gene. In our study, all AFP-producing tumours intensely expressed MAGE-A10 mRNA in the primary lesions. However, the relationship between ATBF1 and MAGE genes and AFP expression is still unknown. Taken together, these findings suggested the aggressive nature of AFP-producing stomach cancer accompanied with MAGE-A10 gene expression.

The function of MAGE family genes and proteins has not been clearly elucidated. In the literature, MAGE-A1, -A2, -A3, -A5, -A6 and -A12 proteins were reported to act as oncoproteins (Yang *et al*, 2007), whereas MAGE-A4 protein was shown to act as an oncosuppressor protein (Peikert *et al*, 2006). MAGE-A3 mRNA expression was an independent poor prognostic marker in adenocarcinoma of the lung (Gure *et al*, 2005). On the other hand, MAGE-A4 mRNA expression correlated with a good clinical outcome in non-small-cell lung cancer (Yoshida *et al*, 2006). Based on our results, MAGE-A10 gene may participate in tumour progression as an oncoprotein, although further analysis is needed.

In spite of significantly high concordance between MAGE-A protein expression by 6C1 and MAGE-A10 gene expression by ISH in our study, 11 cases (29.7%) out of 37 patients with positive MAGE-A10 mRNA were negative for 6C1. Wang *et al* (2004) reported that expression of NY-ESO-1 protein was lower than that of NY-ESO-1 mRNA detected by RT–PCR. It was speculated that immunohistochemical staining could not detect low-level NY-ESO-1 protein expression. Intranuclear substances that are immunohistochemically stained with the 6C1 antibody reflect the MAGE-A10 protein (Rimoldi *et al*, 1999). In fact, our results showed that both the nucleus and cytoplasm of the tumour cells were stained in most 6C1-positive cases. We assume that this discrepancy is mainly caused by the differences in sensitivity and specificity between the two detection systems and target sites of the sequence for a cRNA probe generated by PCR and the 6C1 monoclonal antibody.

The HLA-A2/MAGE-A10_254−262_ peptide is a good target for active immunotherapy of malignant tumours such as melanoma, hepatocellular carcinoma and lung cancer (Dutoit *et al*, 2001; Bricard *et al*, 2005; Groeper *et al*, 2006). Dutoit *et al* (2001) reported that only CTL could lyse MAGE-A10-expressing tumour cells. In our experiments, MAGE-A10 expression was associated with an advanced stage of stomach cancer. High incidence of MAGE-A10 gene expression was observed in both primary and metastatic lesions in hepatic metastasised patients. Thus, the results of this study suggest that MAGE-A10 is a possible candidate target in active immunotherapy for advanced stomach cancer. Clinical trials of dendritic cell vaccine therapy using HLA-A2/MAGE-A10_254−262_ peptide have been conducted (Chianese-Bullock *et al*, 2005; Davis *et al*, 2006). In such an active immunotherapy, the first step is the selection of patients suitable for the treatment. The patients should be selected using immunohistochemical analysis with 6C1 and assessment of MAGE-A10 gene expression using highly sensitive ISH.

In summary, we clarified the role of MAGE-A10 gene expression on tumour progression, especially liver metastasis in patients with stomach cancer. Based on our methods, it could be possible to select the patients for active immunotherapy using the MAGE-A10 peptide.

## Figures and Tables

**Table 1:** Patients' characteristics in each group

	**Group A**	**Group B**	**Group C**
*Age (years)*			
Median (range)	70 (47–90)	63 (43–77)	66 (30–77)
			
*Gender*			
Male/female	37/4	11/4	10/5
			
*Stage*			
IA	1	—	15
IB	1	—	0
II	5	8	—
IIIA	4	4	—
IIIB	6	2	—
IV	24 (23)^a^	1	—
			
Histology			
			
Differentiated^b^	25	4	8
Undifferentiated^c^	16	11	7

a Synchronous liver metastasis.

b tub1, tub2.

c por1, por2, sig.

**Table 2:** Correlation of 6C1 and MAGE-A10 mRNA expression and rate of concordance in each group

	**Group A**	**Group B**	**Group C**
6C1 (+)/MAGE-A10 (+)	25	1	0
6C1 (+)/MAGE-A10 (−)	2	0	0
6C1 (−)/MAGE-A10 (+)	8	3	0
6C1 (−)/MAGE-A10 (−)	6	11	15
Total	41	15	15
Rate of concordance (%)	75.6	80	100

**Table 3:** Expression of 6C1 and MAGE-A10 mRNA in hepatectomised patients

		**Primary**		**Liver**	
**Case**		**6C1**	**MAGE-A10**	**6C1**	**MAGE-A10**
1	M	+	+	+	+
2	M	−	+	−	+
7	S	+	+	+	+
12	S	+	+	+	+
16	M	+	+	+	−
17	S	+	+	+	+
21	S	−	+	+	+
28	S	+	+	+	+
41	S	−	+	−	+

M=metachronous liver metastasis; S=synchronous liver metastasis.
